# Birth and neonatal risk factors associated with neonatal conjunctivitis: a retrospective case–control study

**DOI:** 10.1186/s12887-026-07202-w

**Published:** 2026-06-20

**Authors:** Melike Buşra Atar, Necati Hançerlioğulları, Özdemir Özdemir, Aytekin Tokmak

**Affiliations:** 1 Department of Obstetrics and Gynecology, Ankara Bilkent City Hospital, Ankara, Türkiye; 2 Department of Ophtalmology, Ankara Bilkent City Hospital, Ankara, Türkiye

**Keywords:** Neonatal conjunctivitis, Birth factors, Prematurity, Diabetes mellitus, C-reactive protein, APGAR score

## Abstract

**Background:**

Neonatal conjunctivitis (NC) is a common ocular condition during the neonatal period and may lead to serious complications if not properly managed. Although maternal conditions contribute to its development, birth-related and neonatal factors also play an important role.

**Objective:**

To evaluate birth-related and neonatal factors, along with maternal characteristics, associated with the development of NC.

**Methods:**

This retrospective case–control study was conducted at a tertiary referral center and included neonates born between 34 and 42 weeks of gestation between January 2022 and December 2024. A total of 203 neonates diagnosed with conjunctivitis within the first 28 days of life were compared with 355 randomly selected controls without conjunctivitis from the same source population and study period. Maternal, obstetric, neonatal, and laboratory characteristics were extracted from electronic medical records. Univariable and multivariable logistic regression analyses were performed to identify independent predictors of NC.

**Results:**

A total of 558 neonates were included, comprising 203 cases of NC and 355 controls. Neonates with conjunctivitis were more frequently male and had lower gestational age, higher rates of prematurity, and lower Apgar scores than controls. Maternal age, maternal diabetes, and maternal CRP levels were significantly higher among cases. After adjustment for potential confounders, maternal age (OR 1.08, 95% CI 1.03–1.13), maternal diabetes (OR 2.64, 95% CI 1.17–5.94), male sex (OR 1.84, 95% CI 1.24–2.75), and maternal CRP level (OR 1.11, 95% CI 1.07–1.16) were independently associated with neonatal conjunctivitis (all *p* < 0.05).

**Conclusion:**

In this case–control study, advanced maternal age, maternal diabetes, elevated maternal CRP levels, and male sex were independently associated with NC. Recognition of these maternal and neonatal factors may facilitate earlier identification of at-risk infants and improve postnatal monitoring strategies.

## Introduction

The conjunctiva is a highly specialized, translucent mucosal membrane lining the inner surface of the eyelids and extending over the anterior sclera to the limbus. Beyond serving as a mechanical barrier, it constitutes an integral component of the ocular surface immune system due to its rich vascular, lymphatic, and immune cell networks. Through the production of mucins, antimicrobial peptides, and immunoglobulins, the conjunctiva plays a pivotal role in protecting the neonatal eye from environmental and microbial insults.

Neonatal conjunctivitis (NC), also referred to as ophthalmia neonatorum, is defined as conjunctival inflammation occurring within the first 28 days of life [1]. Despite substantial improvements in obstetric care, antenatal screening programs, and routine postnatal ocular prophylaxis, NC remains one of the most frequently encountered ocular disorders in the neonatal period. Reported incidence rates vary considerably worldwide, ranging from 0.3% to 19%, reflecting differences in screening strategies, microbiological profiles, socioeconomic conditions, and access to perinatal care [2].

Newborns are inherently vulnerable to ocular surface infections due to immunological and anatomical immaturity. Reduced tear production, low levels of secretory immunoglobulin A (sIgA), diminished lysozyme activity, and the relative absence of organized conjunctival-associated lymphoid tissue collectively compromise local immune defense mechanisms [3]. This susceptibility is further influenced by perinatal exposures during vaginal delivery and early postnatal adaptation. NC may result from bacterial or viral pathogens, or from non-infectious inflammatory reactions, particularly in response to topical chemical prophylactic agents administered immediately after birth. The timing of symptom onset often provides important etiological clues; for instance, conjunctivitis presenting within the first hours of life is typically associated with irritative reactions to agents such as silver nitrate [4].

Historically, research has focused predominantly on infectious etiologies—most notably Chlamydia trachomatis and Neisseria gonorrhoeae—given their potential to cause severe ocular morbidity [5]. However, emerging evidence suggests that NC is not solely an infection-driven entity but rather a multifactorial condition shaped by complex interactions between microbial exposure, host immune competence, and perinatal physiology. Increasing attention has been directed toward maternal metabolic disorders, systemic inflammatory status, and perinatal stress responses, all of which may influence neonatal immune function and susceptibility to postnatal infections [6]. Maternal diabetes mellitus, advanced maternal age, and elevated inflammatory markers have been implicated in altering intrauterine immune programming and neonatal inflammatory responses, potentially predisposing neonates to early-life mucosal infections.

Although several studies have characterized microbiological profiles and treatment outcomes of NC, data examining the contribution of maternal obstetric characteristics and perinatal factors remain limited and often fragmented. In particular, the independent effects of maternal metabolic and inflammatory conditions on neonatal conjunctival inflammation have not been comprehensively evaluated in large tertiary care cohorts. This represents a critical knowledge gap, as identification of modifiable maternal and perinatal determinants could facilitate early risk stratification and targeted preventive strategies.

Given the multifactorial nature of NC and the limited evidence addressing maternal and birth-related determinants, a comprehensive evaluation of these factors is warranted. Therefore, the present study aimed to investigate maternal demographic characteristics, obstetric variables, neonatal parameters, and laboratory markers associated with the development of NC in a large tertiary care population, and to identify independent predictors through multivariable analysis.

## Materials and methods

### Study design and setting

This retrospective case–control study was conducted at Ankara Bilkent City Hospital, a high volume tertiary referral center in the capital of Türkiye. The institutional electronic medical records of neonates born between January 1, 2022, and December 31, 2024, were systematically reviewed.

### Study population

Neonates born between 34 + 0 and 42 + 0 weeks of gestation and diagnosed with NC within the first 28 days of life were included in the case group. To ensure comparability in obstetric management, neonatal care practices, and follow-up protocols, the control group consisted of neonates from the same birth cohort and institution who did not exhibit any clinical signs of conjunctivitis during their first 28 postnatal days. Medical records were reviewed on a monthly basis to identify all eligible neonates meeting the predefined inclusion and exclusion criteria. From this pool, controls were selected using systematic random sampling to achieve an approximately two-to-one control-to-case ratio, ensuring balanced representation across the study period and minimizing selection bias. All selected controls had undergone routine neonatal examinations with no documented ocular pathology.

### Inclusion and exclusion criteria

#### Inclusion criteria were

- Singleton pregnancy
- Maternal age between 18 and 45 years
- Delivery at the study institution
- Gestational age between 34 and 42 weeks

#### Exclusion criteria comprised

- Congenital ocular anomalies
- Multiple gestations
- Delivery outside the study center
- Diagnosis of conjunctivitis beyond the neonatal period
- Incomplet or missing missing medical records.
- These criteria were applied uniformly to both groups to reduce selection bias.

### Data collection

Maternal, obstetric, and neonatal data were extracted from standardized electronic medical records. Maternal variables included age, gravida, parity, pre-existing or gestational comorbidities (diabetes mellitus, hypertension, thyroid disorders, asthma, thrombophilia), and laboratory parameters obtained prior to delivery.

Complete blood count (CBC) parameters, including hemoglobin, hematocrit, leukocyte count with differential, and platelet count, were measured using an automated hematology analyzer. Biochemical parameters, including glucose, creatinine, aspartate aminotransferase, and alanine aminotransferase, were analyzed using standard automated clinical chemistry analyzers in the hospital laboratory. Maternal C-reactive protein (CRP) levels were assessed using a quantitative immunoturbidimetric assay and reported as continuous values (mg/dL). Urinalysis was performed using automated urine analyzers, with microscopic examination conducted when indicated.

Obstetric variables included mode of delivery (vaginal or cesarean), status and duration of membrane rupture, intrapartum antibiotic administration, induction of labor, and antenatal corticosteroid exposure. Neonatal variables included sex, gestational age, birth weight, birth length, head circumference, 1- and 5-minute Apgar scores, need for neonatal intensive care unit (NICU) admission, and length of hospital stay. As part of the institutional protocol, all neonates received routine ocular prophylaxis with 2.5% povidone–iodine solution immediately after birth.

### Clinical diagnosis and classification of conjonctivitis

The diagnosis of NC was established by experienced pediatric ophthalmologist (OO) based on documented clinical findings, including conjunctival hyperemia, ocular discharge, and eyelid edema within the first 28 days of life. To minimize interobserver variability, all clinical classifications were performed by the same experienced pediatric ophthalmologist. Cases were further classified as either infectious or noninfectious according to a standardized clinical algorithm. Noninfectious conjunctivitis, often chemical or mechanical, was considered when symptoms manifested within the first 24–48 h after birth, particularly following prophylactic ocular treatment, and was characterized by mild conjunctival injection and watery discharge with rapid resolution under supportive care. Conversely, infectious conjunctivitis was suspected when symptoms developed after 48 h of life, typically between days 3 and 14, and was associated with purulent discharge, conjunctival hyperemia, eyelid edema, and, in some cases, bilateral involvement. Classification was additionally supported by clinical course and risk factors, such as prolonged rupture of membranes and maternal genital infection history. Cases presumed to be infectious were treated with topical antibiotics according to institutional neonatal ophthalmology protocols and followed until complete clinical resolution, whereas noninfectious cases received supportive management only. Reflecting routine neonatal ophthalmology practice where diagnosis is primarily clinical and culture yield is often limited, conjunctival microbiological cultures were not routinely obtained. Instead, they were reserved for selected cases with atypical presentation or inadequate response to empirical therapy, and the results from this limited number of infants were interpreted in conjunction with clinical findings.

### Outcome definition

The primary outcome was the occurrence of NC within the first 28 days of life. Only clinically confirmed cases documented by pediatric ophthalmologists were included to ensure diagnostic consistency.

### Statistical analysis

Statistical analyses were performed using standard statistical software. The distribution of continuous variables was assessed for normality using visual (histograms, Q–Q plots) and analytical methods. Normally distributed variables were expressed as mean ± standard deviation (SD), whereas non-normally distributed variables were presented as median and interquartile range (IQR). Categorical variables were summarized as frequencies and percentages. Between-group comparisons were conducted using Student’s t-test for normally distributed continuous variables, the Mann–Whitney U test for non-normally distributed variables, and the Chi-square or Fisher’s exact test for categorical variables, as appropriate. To identify independent predictors of NC, variables demonstrating a p-value < 0.10 in univariate analyses were entered into a multivariate logistic regression model using a backward stepwise approach. Odds ratios (ORs) with 95% confidence intervals (CIs) were calculated. Multicollinearity was assessed prior to model construction. Confounding was addressed through multivariate adjustment rather than matching, thereby allowing simultaneous evaluation of multiple maternal and neonatal risk factors. Model fit was assessed using appropriate goodness-of-fit statistics. A two-sided p-value < 0.05 was considered statistically significant.

## Results

**Fig. 1:**
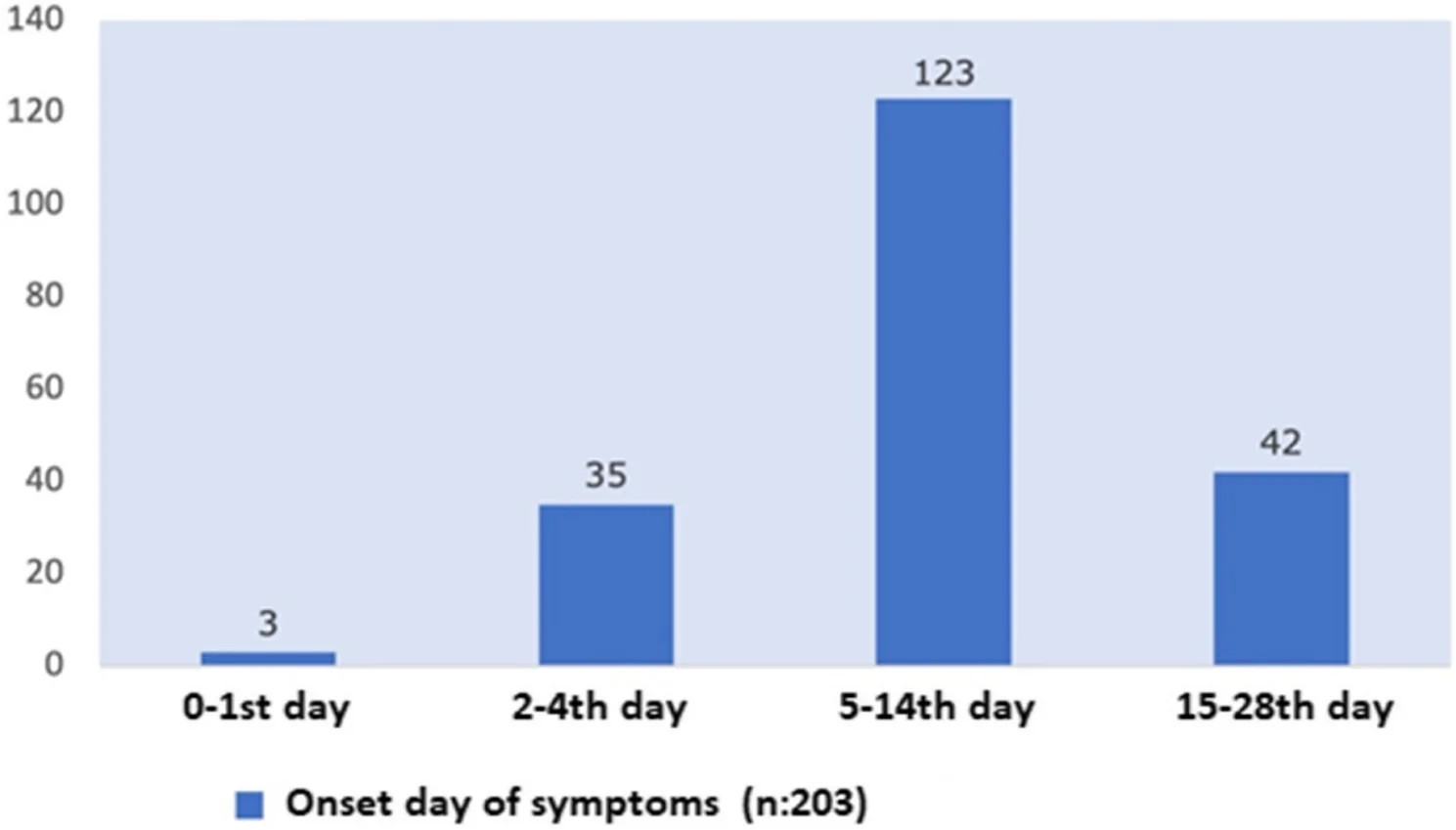
Neonatal conjuncti̇vi̇ti̇s by day of onset after bi̇rth

A total of 558 neonates met the inclusion criteria, including 203 diagnosed with neonatal conjunctivitis (NC) and 355 controls. Among neonates diagnosed with NC (*n* = 203), symptom onset occurred within the first 24 h of life in 3 cases (1.5%), between days 1–4 in 35 cases (17.2%), between days 5–14 in 123 cases (60.6%), and after the first 2 weeks of life in 42 cases (20.7%). The distribution of neonatal conjunctivitis cases by day of symptom onset after birth is presented in Fig. 1. Maternal age was significantly higher in the NC group compared with controls (29 ± 5 vs. 27 ± 5 years, *p* < 0.001). The prevalence of maternal diabetes mellitus was also significantly greater among neonates who developed NC (11% vs. 3%, *p* < 0.001) (Table 1).

**Table 1:** Baseline maternal characteristics of the study population

Variable	Total (*n* = 558)	Conjunctivitis (*n* = 203)	Control (*n* = 355)	*p*-value
Maternal age (years)	27.7 ± 5.0	29.3 ± 5.3	26.8 ± 4.6	< 0.001
Gravida, median	2 (1–3)	2 (1–3)	2 (1–3)	0.003
Parity,	2 (1–2)	2 (1–3)	2 (1–2)	0.003
Diabetes mellitus	34 (6%)	22 (11%)	12 (3%)	< 0.001
Hypertension	8 (1%)	7 (3%)	1 (0.3%)	0.002
Thyroid disorder	74 (13%)	34 (17%)	40 (11%)	0.066
Asthma	8 (1%)	5 (3%)	3 (1%)	0.122
Thrombophilia	8 (1%)	7 (3%)	1 (0.3%)	0.002
Intrahepatic cholestasis	3 (0.5%)	3 (1.5%)	0	0.052
Renal disease	3 (0.5%)	3 (1.5%)	0	0.052

Values are presented as mean ± standard deviation, median (25^th^-75^th^ percentile), or number (%). A *p* value<0.05 is statistically significant

Among affected mothers, gestational diabetes mellitus (GDM) accounted for the majority of cases in both groups. In the conjunctivitis group, 13 mothers had diet-controlled GDM and 6 had insulin-treated GDM, whereas in the control group these numbers were 10 and 2, respectively. Type 1 and type 2 diabetes were observed only in the conjunctivitis group (*n* = 2 and *n* = 1, respectively) and were not present among controls.

With respect to neonatal characteristics, male sex was more common in the NC group (60% vs. 48%, p:0.011). Gestational age was significantly lower among affected neonates, and the rate of preterm birth was higher compared to controls (14% vs. 8%, p:0.023). Both 1st-minute and 5th-minute Apgar scores were significantly lower in neonates with NC. Birth length was shorter in the NC group, whereas birth weight and head circumference did not differ significantly between groups (Table 2). Neonates with NC had a higher rate of NICU admission (13% vs. 6%, p:0.003). However, the duration of hospitalization was comparable between groups.

**Table 2:** Comparison of maternal and neonatal characteristics of groups that developed neonatal conjunctivitis and those that did not (control)

Variables	Case (*n* = 203)	Control (*n* = 355)	*P* value
*Obstetrics characteristics*			
Mode of Delivery			0.117
Vaginal delivery with episiotomy	48 (%24)	115 (%32)	
Vaginal delivery without episiotomy	47 (%23)	73 (%21)	
Water birth	2 (%1)	7 (%2)	
Operative vaginal delivery	1 (%0,5)	5 (%1)	
Cesarean section	105 (%52)	155 (%44)	
Cesarean Section Group (*n* = 260)			0.191
Emergency	79 (%75)	127 (%82)	
Elective	26 (%25)	28 (%18)	
Rupture of Membranes (*n* = 114)	35 (%17)	79 (%22)	0.158
Duration of Membrane Rupture			0.248
< 18 h	26 (%74)	66 (%83)	
≥18 h	9 (%26)	13 (%17)	
Antibiotic use (including surgical prophylaxis)	112 (%55)	179 (%50)	0.280
Antibiotic use (excluding cesarean section prophylaxis)	18 (%9)	37 (%10)	0.553
Labor induction	62 (%30)	125 (%35)	0.261
Method of Labor Induction (*n* = 187)			0.346
Prostaglandin E2 (Propess)	7 (%11)	13 (%10)	
Oxytocin infusion	51 (%82)	95 (%76)	
Combined method	4 (%7)	17 (%14)	
Neonatal Characteristics			
Neonatal sex			0.011
Male	121 (%60)	172 (%48)	
Female	82 (%40)	183 (%52)	
Birth week	38.5 ± 1.6	38.9 ± 1.4	0.011
Preterm birth	29 (%14)	29 (%8)	0.023
APGAR scoe 1st mn.	7.5 ± 0.6	7.6 ± 0.5	0.021
APGAR skor 5th mn.	8.9 ± 0.5	9.0 ± 0.4	0.004
Birth weight (gram)	3181 ± 465	3245 ± 423	0.103
Head circumference (cm)	34.5 ± 1.4	34.6 ± 1.4	0.474
Length (cm)	49.6 ± 2.0	50.0 ± 1.8	0.011
Neonatal intensive care unit (NICU) admission (*n* = 48)	27 (%13)	21 (%6)	0.003
Length of NICU stay	4.0 (2.2–4.7)	4.0 (2.0–5.0)	0.585
Antenatal betamethasone administration (*n* = 58)	26 (%13)	32 (%9)	0.158
Betamethasone dosage			0.291
Single dose	11 (%42)	18 (%56)	
Two doses	15 (%58)	14 (%44)	
Betamethasone administration within the last 14 days (*n* = 58)	19 (%73)	20 (%62)	0.393
Day of symptom onset (*n* = 203)	8.0 (5.0–13.0)	.	NA

Values are presented as mean ± standard deviation, median (25^th^ -75^th^ percentile), or number (%). A *p* value < 0.05 is statistically significant

Regarding maternal laboratory parameters, C-reactive protein (CRP) and serum creatinine levels were significantly higher in mothers of neonates with NC, while other laboratory variables did not demonstrate statistically significant differences (Table 3).

**Table 3:** Comparison of laboratory parameters of neonatal conjunctivitis developing and non-developing (control) groups

Variables		Case (*n* = 203)	Control (*n* = 355)	*p*-value
*Blood parameters*				
Hemoglobin (g/dL)		12.0 ± 1.2	11.9 ± 1.3	0.086
Hematocrit (%)		36.9 ± 3.3	36.5 ± 4.7	0.167
Platelet count (×10³/µL)		244 ± 70	250 ± 70	0.306
White blood cell count (×10³/µL)		9.97 (8.60-11.63)	9.99 (8.53–11.42)	0.809
Neutrophil count (×10³/µL)		7.31 (6.20–8.93)	7.35 (6.05–8.69)	0.786
Lymphocyte count (×10³/µL)		1.73 (1.49–2.09)	1.82 (1.53–2.16)	0.253
Neutrophil-to-lymphocyte ratio		4.1 (3.3–5.1)	4.0 (3.2–5.1)	0.636
C-reactive protein (mg/dL)		8.6 (5.3–13.0)	6.2 (3.3–9.6)	< 0.001
Thyroid-stimulating hormone (mIU/mL)		1.8 (1.2–2.7)	1.9 (1.4–2.7)	0.557
Glucose (mg/dL)		82 (71–89)	80 (71–88)	0.395
Creatinine (mg/dL)		0.52 ± 0.10	0.50 ± 0.10	0.038
Aspartate aminotransferase (IU/L)		17 (13–22)	17 (13–21)	0.859
Alanine aminotransferase (IU/L)		13 (11–17)	13 (10–16)	0.370
*Urinalysis Parameters*				
Proteinuria (> trace)		24 (%12)	43 (%12)	0.919
Nitrite		0	2 (%1)	0.536
Leukocytes (≥ 5 per high-power field)		102 (%50)	166 (%47)	0.428
Bacteria (moderate/abundant)		15 (%7)	35 (%10)	0.326

Values are presented as mean ± standard deviation, median (25^th^ -75^th^ percentile), or number (%). A *p* value < 0.05 is statistically significant

In multivariable logistic regression analysis, advanced maternal age, maternal diabetes mellitus, male sex, and elevated maternal CRP levels remained independently associated with the development of NC (Table 4).

**Table 4:** Univariate and multivariate logistic regression analysis of neonatal conjunctivitis development

Variables	Univariate			Multivariate		
	OR	95% CI	p-value	OR	95% CI	*p*-value
Age (years)	1.11	1.07–1.15	< 0.001	1.08	1.03–1.13	0.001
Gravida	1.28	1.10–1.50	0.001	1.14	0.93–1.41	0.210
Diabetes mellitus	3.47	1.68–7.18	0.001	2.64	1.17–5.94	0.020
Thyroid disorder	1.58	0.97–2.57	0.068	1.61	0.91–2.86	0.100
Mode of delivery	1.10	0.99–1.21	0.058	1.21	0.66–2.23	0.473
Rupture of membranes	0.73	0.47–1.13	0.159	.	.	.
Duration of membrane rupture	1.76	0.67–4.61	0.251	.	.	.
Antibiotic use	1.21	0.86–1.71	0.280	.	.	.
Labor induction	0.81	0.56–1.17	0.261	.	.	.
Neonatal sex (male)	1.57	1.10–2.23	0.011	1.84	1.24–2.75	0.003
Neonatal length	0.89	0.81–0.97	0.009	0.90	0.80–1.01	0.085
Gestational age	0.86	0.76–0.96	0.009	1.04	0.86–1.27	0.669
Preterm birth	1.87	1.08–3.23	0.024	1.19	0.49–2.89	0.704
5-minute APGAR score	0.55	0.37–0.81	0.003	0.64	0.41–1.01	0.051
NICU admission	2.44	1.34–4.44	0.004	1.75	0.82–3.72	0.147
Length of NICU stay	1.01	0.86–1.18	0.894	.	.	.
Antenatal betamethasone administration	1.48	0.86–2.57	0.160	.	.	.
White blood cell count	0.99	0.93–1.07	0.925	.	.	.
C-reactive protein	1.12	1.08–1.16	< 0.001	1.11	1.07–1.16	< 0.001
Creatinine	6.51	1.12–37.66	0.037	5.29	0.66–42.12	0.115
Bacteriuria	1.37	0.73–2.58	0.327	.	.	.

*OR* Odds ratio, *CI* Confidence interval

A *p* value < 0.05 is statistically significant

Conjunctival cultures were obtained in a limited number of patients (*n* = 8) based on clinical indications. Bacterial growth was detected in six cultures, including Staphylococcus epidermidis (*n* = 2), Pseudomonas aeruginosa (*n* = 1), Streptococcus mitis/oralis (*n* = 1), Serratia marcescens (*n* = 1), and Moraxella spp. (*n* = 1), while two cultures showed no growth.

## Discussion

In this large tertiary care cohort, NC was independently associated with advanced maternal age, maternal diabetes mellitus, male sex, and elevated maternal CRP levels at delivery. These findings support the concept that NC is not solely a consequence of perinatal microbial exposure but rather reflects a multifactorial susceptibility profile shaped by maternal metabolic, inflammatory, and neonatal biological factors.

Advanced maternal age emerged as an independent predictor of NC. Increasing maternal age has consistently been associated with adverse perinatal outcomes, including prematurity, low birth weight, and heightened neonatal infection risk [7–9]. In our cohort, each one-year increase in maternal age was associated with an approximately 8% increase in NC risk, even after multivariate adjustment. This observation suggests that maternal age may act as a composite marker of obstetric complexity, immune modulation, or placental alterations that collectively influence neonatal vulnerability. Given the global trend toward delayed childbearing, this association carries increasing public health relevance.

Maternal diabetes mellitus was another strong independent determinant, conferring a 2.6-fold increase in NC risk. Beyond glycemic dysregulation, diabetes is characterized by chronic low-grade inflammation and immune dysfunction, both of which may alter the intrauterine environment and fetal immune programming [10]. Experimental and translational studies have demonstrated that gestational diabetes enhances placental inflammation through activation of autophagy-related and TLR4/MyD88/NF-κB signaling pathways [11], while transcriptomic analyses indicate altered fetal monocyte activation and pro-inflammatory phenotypes [12]. Such immunologic modulation may impair neonatal mucosal defense mechanisms, including conjunctival immune responses, thereby increasing susceptibility to early-life infections. Our findings align with this mechanistic framework and highlight maternal metabolic status as a clinically relevant determinant of neonatal ocular infection risk.

Sex-based differences in immune maturation may further explain the observed association between male sex and NC. Increasing evidence indicates that immune dimorphism is established during fetal development. Fairweather et al. described sex-specific immune regulation influenced by sex hormones, X chromosome–linked immune genes, and epigenetic mechanisms [13]. In contrast to the heightened autoimmune predisposition observed in females, male neonates may exhibit relatively delayed immune maturation, potentially increasing vulnerability to infectious conditions. Similarly, Muenchhoff and Goulder reported that sex-related disparities in T-helper cell responses are detectable from the fetal stage and may contribute to more severe infectious outcomes in male infants [14]. Although the underlying mechanism remains unclear, male infants have been reported to exhibit greater susceptibility to infectious and inflammatory conditions during the neonatal period. As suggested in previous studies, proposed explanations include sex-related differences in innate and adaptive immune responses, hormonal influences, and variations in early mucosal immune regulation. Consistent with these observations, male sex was independently associated with neonatal conjunctivitis in our cohort.

Prematurity and lower gestational age were associated with NC in univariate analyses. Preterm infants exhibit impaired epithelial barrier integrity, reduced tear production, and diminished local antimicrobial activity, all of which may compromise conjunctival defense [15, 16]. Although gestational age did not retain independent significance in multivariate modeling, its association in unadjusted analyses suggests that prematurity may operate through intermediate pathways linked to systemic vulnerability. In parallel, lower Apgar scores were observed among affected neonates, reflecting perinatal stress and hypoxia. Large population-based data have demonstrated that even modest reductions in Apgar scores correlate with increased morbidity and mortality [17]. However, the absence of independent predictive value in our model indicates that Apgar scores likely represent global neonatal fragility rather than a direct causal factor for NC.

Neonatal intensive care unit (NICU) admission was more frequent among neonates with NC in univariate analyses. Previous studies have shown that low birth weight, invasive respiratory support, and ophthalmic screening procedures increase the risk of nosocomial conjunctivitis in NICU settings [18]. In our analysis, however, NICU admission did not remain independently associated with NC after adjustment, suggesting that intensive care exposure may function as a surrogate for prematurity or underlying morbidity rather than as a direct etiological factor. Nevertheless, strict infection control practices remain essential in high-risk neonatal environments.

A particularly noteworthy finding of our study is the independent association between elevated maternal CRP levels at delivery and NC. CRP is widely used as a biomarker of systemic inflammation and has been extensively investigated in neonatal sepsis [19]. However, data specifically linking maternal inflammatory markers to neonatal conjunctival disease are scarce. The observed association suggests that maternal inflammatory milieu at the time of delivery may influence neonatal immune competence or microbial colonization dynamics. Although the discriminatory performance of maternal CRP alone was moderate, its incorporation into multifactorial risk assessment models may enhance early identification of at-risk neonates.

Mild elevations in maternal serum creatinine were also observed in the NC group, though not independently predictive. While direct associations between maternal renal function and NC have not been previously described, renal dysfunction during pregnancy is often linked to systemic inflammation and metabolic disorders [20]. Therefore, creatinine elevation may reflect broader maternal physiological stress rather than a specific pathogenic pathway. Prospective studies are needed to clarify whether maternal renal parameters contribute independently to neonatal infectious risk.

Contrary to some reports suggesting that prolonged rupture of membranes increases NC risk [21–23], we did not observe a significant association. Similarly, mode of delivery was not independently related to NC, despite meta-analytic evidence indicating higher incidence following vaginal birth [24]. These discrepancies may reflect effective intrapartum antibiotic protocols, standardized ocular prophylaxis with povidone–iodine, and stringent infection control measures implemented at our institution.

Taken together, our findings indicate that NC arises within a multifactorial framework in which maternal metabolic and inflammatory conditions intersect with neonatal biological susceptibility. Similar composite risk profiles have been described in neonatal sepsis and pneumonia [25, 26], suggesting that NC may represent a localized manifestation of broader immunological vulnerability rather than an isolated ocular process.

The distribution of symptom onset demonstrated a clear peak between the 5th and 14th postnatal days, accounting for the majority of cases. This pattern is consistent with infectious neonatal conjunctivitis, which typically presents after the immediate postnatal period. A smaller proportion of cases occurred within the first 48 h of life, which may reflect chemical or early irritative conjunctivitis related to perinatal prophylaxis or mechanical factors. The relatively lower frequency of late-onset cases (15–28 days) suggests that most clinically significant presentations occur within the first two weeks of life, highlighting the importance of close early neonatal surveillance.

Although microbiological cultures were not routinely performed, the organisms identified in culture-positive cases were broadly consistent with pathogens previously reported in tertiary-care neonatal units, including coagulase-negative staphylococci, Pseudomonas aeruginosa, and Serratia marcescens [27]. However, the small number of cultures precludes firm conclusions regarding the microbiological epidemiology of neonatal conjunctivitis in our cohort.

The strengths of this study include its relatively large sample size, uniform diagnostic criteria established by an experienced pediatric ophthalmologists, and comprehensive multivariable modeling. However, several limitations warrant consideration. The retrospective design precludes causal inference. Microbiological data were limited, as routine repeat conjunctival cultures were not systematically performed. Information regarding maternal genital colonization, subclinical infections, and antibiotic exposure beyond intrapartum administration was incomplete. Finally, the single-center design and the relatively limited follow-up duration for the control group may restrict the external generalizability of our findings. However, because all participants originated from the same hospital population and uniform follow-up system, the potential risk of surveillance bias was significantly minimized.

## Conclusion

Advanced maternal age, maternal diabetes mellitus, male sex, and elevated maternal CRP levels are independent predictors of neonatal conjunctivitis. These findings underscore the importance of integrated maternal–neonatal risk assessment and suggest that systemic maternal conditions contribute to neonatal ocular infection susceptibility. Prospective, multicenter studies incorporating detailed microbiological and immunological profiling are needed to further elucidate underlying mechanisms and refine preventive strategies.

### Clinical implications

From a clinical standpoint, incorporation of maternal age, diabetic status, and inflammatory markers into perinatal risk assessment may facilitate early surveillance strategies for neonates at increased risk. Recognition of male sex as a biological susceptibility factor further supports individualized monitoring. Such stratified approaches may enable earlier diagnosis and timely management, potentially reducing short- and long-term ocular morbidity.

## Data Availability

The data sets may be made available from the corresponding author upon reasonable request and with permission from the Institutional Ethics Committee of Ankara Bilkent City Hospital.

## Abbreviations

NC: Neonatal Conjunctivitis
CRP: C-Reactive Protein
NICU: Neonatal Intensive Care Unit
GDM: Gestational Diabetes Mellitus
